# Cardiovascular Disease and SARS-CoV-2: the Role of Host Immune Response Versus Direct Viral Injury

**DOI:** 10.3390/ijms21218141

**Published:** 2020-10-30

**Authors:** Federico Biscetti, Maria Margherita Rando, Elisabetta Nardella, Andrea Leonardo Cecchini, Piergiorgio Bruno, Raffaele Landolfi, Andrea Flex

**Affiliations:** 1 Fondazione Policlinico Universitario A. Gemelli IRCCS, 00168 Roma, Italy; piergiorgio.bruno@policlinicogemelli.it (P.B.); raffaele.landolfi@unicatt.it (R.L.); andrea.flex@unicatt.it (A.F.); 2 Internal Medicine and Vascular Diseases Unit, 00168 Roma, Italy; m.margheritarando@gmail.com (M.M.R.); elisabetta.nardella@gmail.com (E.N.); alcech92@gmail.com (A.L.C.); 3 Laboratory of Vascular Biology and Genetics, Department of Translational Medicine and Surgery, 00168 Roma, Italy; 4 Cardiac Surgery Unit, 00168 Roma, Italy; 5 Università Cattolica del Sacro Cuore, 00168 Roma, Italy

**Keywords:** cardiovascular diseases, SARS-CoV-2

## Abstract

The 2019 novel coronavirus [2019-nCoV], which started to spread from December 2019 onwards, caused a global pandemic. Besides being responsible for the severe acute respiratory syndrome 2 [SARS-CoV-2], the virus can affect other organs causing various symptoms. A close relationship between SARS-CoV-2 and the cardiovascular system has been shown, demonstrating an epidemiological linkage between SARS-CoV-2 and cardiac injury. There are emerging data regarding possible direct myocardial damage by 2019-nCoV. In this review, the most important available evidences will be discussed to clarify the precise mechanisms of cardiovascular injury in SARS-CoV-2 patients, even if further researches are needed.

## 1. Introduction

In December 2019, several pneumonia cases of unknown origin were detected in Wuhan, Hubei province, China and in January 2020, a new coronavirus of zoonotic origin named 2019 novel corononavirus [2019-nCoV] was isolated [1,2].

On March 11, 2020 the World Health Organization declared the SARS-CoV-2 a pandemic, with 17,660,523 confirmed cases and 680,894 deaths worldwide on August 2, 2020 [3,4]. Coronaviruses are enveloped, non-segmented, positive-sense RNA viruses widely distributed among animals and humans [5,6].

In the past, two coronavirus have crossed species barriers causing severe pneumonia in humans, known as severe acute respiratory syndrome coronavirus [SARS-CoV] in 2003, with a mortality rate of 10%, and Middle East respiratory syndrome coronavirus [MERS-CoV] in 2012, with a mortality rate of 37% [6,7]. Four low-pathogenic coronavirues―HCoV-229E, HCoV-OC43, HCoV-NL63, and HCoV-HKU1―exist, which cause common cold symptoms in immunocompetent individuals [7].

SARS-CoV-2 is characterized by a wide spectrum of symptoms, such as fever, cough, sore throat, headache, conjunctival and nasal congestion, fatigue, myalgia and arthralgia [1]. Moreover, lymphocytopenia, leukopenia and thrombocytopenia are common among hospitalized patients [8]. Several individuals show increased levels of C-reactive protein, alanine aminotransferase, aspartate aminotransferase, creatine kinase and D-dimer [1]. Chest Computed tomography [CT]-scans demonstrate ground-glass opacity and bilateral patchy shadowing as a common pattern [1]. More severe cases develop interstitial pneumonia and severe acute respiratory distress syndrome [ARDS], which reflects the characteristic pulmonary tropism of 2019-nCoV [9]. Furthermore, the severity of the illness seems to be directly correlated with the host immune response to the infection. However, other organs, in particular the cardiovascular system, may be affected. In addition, patients with pre-existing cardiovascular diseases, diabetes, hypertension, chronic pulmonary disease and cancer are at higher risk for severe complications and death [10].

In this review, we will report the relationship between 2019-nCoV infection and the cardiovascular system. In addition, cardiovascular manifestations of coronavirus disease 2019 [COVID-19] will be discussed.

## 2. SARS-CoV-2 and the Role of the Angiotensin-Converting Enzyme 2

The 2019-nCoV belongs to the family of coronaviruses, a group of enveloped, positive strand, RNA viruses, widely spread among mammals, which are able to cross species barrier infecting humans [6,11,12]. The coronavirus surface is characterized by transmembrane spike [S] glycoproteins, which mediates the entry of the virus into cells [7,12]. S glycoproteins are composed of two subunits: the S1 subunit, which binds host cell receptors and the S2 subunit, which is responsible for viral and cellular membrane fusion [7]. Moreover, entry into the cells requires S protein priming by serine protease TMPRSS2 [12]. Similar to SARS-CoV, angiotensin-converting enzyme [ACE] 2 has been identified as the cellular receptor of 2019-nCoV. The ACE 2 gene is located on the chromosome Xp22, which is expressed in the lung, heart, endothelium, gut, liver and kidneys [13,14,15]. ACE 2 acts as a zinc metallopeptidase and has a role in the renin-angiotensin system [RAS] as well as in cardiovascular protection, counterbalancing the role of ACE [16]. ACE 2 hydrolyses angiotensin [Ang] I, generating Ang 1-9, and Ang II generating Ang 1-7 leading to vasodilatory, antifibrotic, antiproliferative and anti-inflammatory effects [15,17,18,19].

In particular, ACE 2 knockdown in mice increased proinflammatory cytokine levels and pro-fibrosis gene expression, while administration of recombinant ACE 2 mitigated Ang II action, reducing reactive oxygen species [ROS], tumor growth factor [TGF]-β1, fibronectin and collagen levels [15].

Given the spread of SARS-CoV-2, concerns exist about the role of pharmacological RAS blockade agents, and in particular, ACE inhibitors [ACEi] and angiotensin receptor blockers [ARBs]. In fact, viral load was reduced in ACE 2 knockdown mice with SARS-CoV infection [16,20], while different studies demonstrated that ACEi and ARBs increases levels of cardiac, renal, systemic ACE 2 [17]. Therefore, ACEi and ARBs may facilitate 2019-nCoV infection in patients with cardiovascular disease, hypertension and diabetes [17].

Interestingly, mice with SARS-CoV infection showed reduced levels of ACE 2 and increased Ang II levels in lungs [21]. This correlates with severe lung injury, which is related to increased vascular permeability [16]. In fact, Ang II enhances inflammatory cytokines production, alveolar cells apoptosis and fibrosis in response to hypoxia [18]. Moreover, decreased ACE 2 levels upregulate inflammatory cytokines worsening outcomes after myocardial infarction, while overexpression of ACE 2 ameliorates cardiac remodeling [15]. It has even been shown even that recombinant ACE 2 ameliorated lung injury in avian H5N1 and H7N9 influenza [16,22].

ACE 2 activity regulates even blood pressure, which reduces Ang II levels and increases Ang 1-7 levels [15,18]. In addition, it reduces vascular inflammation, which plays a role in atherosclerosis [15]. The 2019-nCoV seems to downregulate ACE 2 expression and Ang 1-7 production, leading to increased levels of Ang II exacerbating lung injury [17]. Ang II binds two different receptors: ATR [Angiotensin II receptor type] 1 and ATR2. ATR1 enhances inflammation, fibrosis and formation of ROS, while ATR2 plays an anti-inflammatory role [18]. With the blockage of AT1R by an ARB, Ang II could act on AT2R with a positive effect on pulmonary injury [18]. Moreover, ACEi reduces Ang II levels and its effect on ATR1 and ATR2 [18]. Ang II seems to induce ACE 2 lysosomal internalization with decreased tissue expression. Administering losartan stimulates interaction and stabilization of ACE 2 with AT1R. Thus, ARBs could reduce viral entry to cells, decreasing internalization of ACE 2 and availability of their binding sites [14]. In addition, Ferrario et al. showed that lisinopril, enalapril and losartan reduced Ang II levels, increased ACE 2 RNA and Ang 1-7 expression [23]. To date, no evidence confirms that patients treated with RAS blockers have a better prognosis [13]. Furthermore, no evidence proves that ACEi and ARBs have an association with SARS-CoV-2 and that they could affect the susceptibility to the infection [24]. Therefore, ACEi and ARBs should not be interrupted in case of SARS-CoV-2.

**Figure 1:**
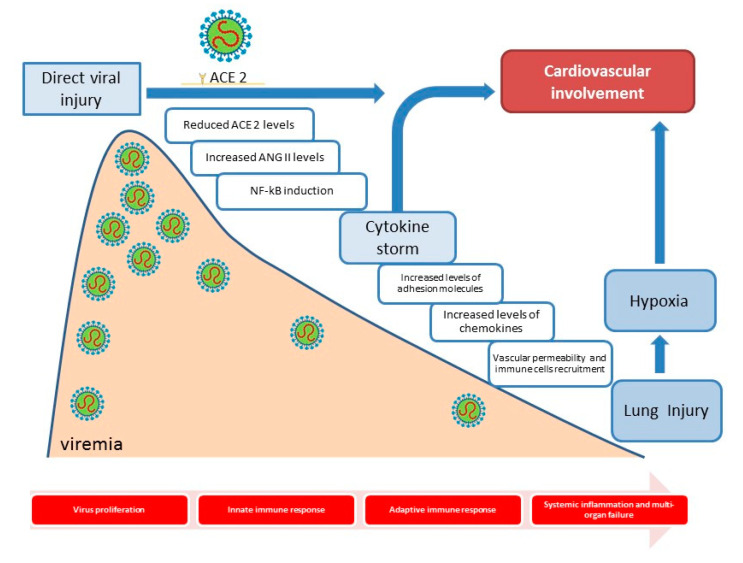
Cardiovascular involvement of SARS-CoV-2 during the various stages of the disease.

Similar to SARS-CoV, SARS-CoV-2 leads to viral-mediated ACE 2 downregulation, increasing Ang II levels. This coincides with the early phase of 2019-nCoV infection, characterized by virus replication in lung tissue and innate immunity activation. In fact, Ang II, acting on AT1R, induces NF-κB [25]. NF-κB promotes expression of several cytokines, in particular interleukin [IL]-1, IL-2, IL-6, IL-8, IL-12, tumor necrosis factor [TNF]-α, chemokines [monocyte chemoattractant protein-1 -MCP-1] and adhesion molecules [P- and L-selectin, VCAM-1, ICAM-1] [26], favouring increased vascular permeability and inflammatory cell recruitment, which results in lung injury and hypoxia. In SARS-CoV, in fact, increased IL-1β, IL-8, IL-6, CXC-chemokine ligand 10 [CXCL10] and CC-chemokine ligand 2 [CCL2] levels correlated with progression of acute respiratory distress syndrome [27]. Moreover, AT1R activation seems to stimulate NAD[P]H oxidase and reactive oxygen species [ROS] production [28]. Interestingly, 2019-nCoV infection seems to cause apoptosis and pyroptosis in macrophages and lymphocytes [29], which explains peripheral blood lymphopenia seen in SARS-CoV-2 [1]. With progression of inflammatory response, viral replication decreases with activation of adaptive immunity and formation of neutralizing antibodies. This phase coincides with respiratory deterioration leading to acute respiratory disease [29] [Figure 1]. Interestingly, a work by Long et al. reported higher IgG and IgM titres in patients with severe SARS-CoV-2 [3]. Some patients develop systemic inflammation, which leads to multi-organ injury [30]. However, further data are needed to demonstrate underlying SARS-CoV-2 pathogenesis mechanisms.

## 3. SARS-CoV-2, Immune System and Immunogenetics

Understanding the role of immune system in SARS-CoV-2 is crucial to clarify the pathogenesis of the disease. SARS-CoV-2 is characterized in the early phase by activation of innate immunity leading to activation of NF-κB with the beginning of a cytokines cascade [31]. Subsequently, adaptative immune response with CD4+ and CD8+ T cells activation enhances the inflammatory process. CD8+ cells are responsible of interferon [IFN]- γ, TNF-α, IL-2, perforin and granzyme B production, which mediate viral clearance [31]. CD4+ cells are essential to the production of antibodies by activating T-dependent B cells. Moreover, the later phase of SARS-CoV-2 is characterized by decreased levels of Th1 cells, increased levels of Th2 cells and Th17 cells, which promote neutrophils and monocytes recruitment, increasing tissue injury [31]. However, the contribution of immune system to SARS-CoV-2 is not fully understood. Manifestation of 2019-nCoV infection are various and of varying severity among affected people. This could be due to a variability in genetic substrate. Although not yet proven, polymorphisms in cytokines genes may be correlated to SARS-CoV-2 severity as shown for SARS-CoV [32]. Moreover, polymorphism in human leucocyte antigens [HLA] has already been correlated to SARS-CoV [33,34,35]. Regarding SARS-CoV-2, Poulton et al. reported an association between HLA- DQB1*06 and the infection [36]. Nguyen et al. demonstrated that HLA-B*46:01 is associated with a major vulnerability to SARS-CoV-2 [37]. In addition, Wang et al. showed that the number of CD4+ T cells, CD8+ T cells, and B cells is reduced in case of severe disease and HLA-DR and CD45RO expression on CD4+ T cells and CD8+ T cells is increased. They showed also that CD8+ T cells and CD4+ T cells producing IFN-γ increase in severe illness [38]. Additional works are needed to identify other genes involved in the infection and its manifestation.

## 4. SARS-CoV-2 and the Cardiovascular System

### 4.1. SARS-CoV-2 and Cardiovascular Comorbidities

A linkage between SARS-CoV-2 and cardiovascular diseases has been demonstrated. In particular, the infection may exacerbate pre-existing or induce new cardiovascular conditions [8]. Patients with a cardiovascular comorbidity may be more susceptible to 2019-nCoV induced heart injury. In particular, Shi et al. showed that patients with cardiac injury had a history of coronary heart disease [30%] and hypertension [60%] [39].

Moreover, SARS-CoV-2 is more aggressive in older age and in patients with comorbidities, such as diabetes, hypertension, ischemic heart disease, cancer, atrial fibrillation, dementia and history of stroke, and the risk of severe disease, requiring intensive care or causing higher mortality rates, is higher [1,6,40,41,42,43]. In particular, a retrospective multicenter cohort study by Zhou et al., including 191 patients, showed that hypertension [30%], diabetes [19%] and coronary heart disease [8%] were the most common comorbidities among patients with SARS-CoV-2 and more than half of the patients, who died, had increased high sensitivity cardiac troponin I [40]. Similarly, Wu et al. demonstrated that hypertension and diabetes were the most common comorbidities, affecting respectively 19.4% and 10.9% of patients [43]. A meta-analysis by Zuin et al. showed that hypertension was the most frequent cardiovascular comorbidity and patients with hypertension had a higher mortality risk compared with normotensive patients [44]. As mentioned, even diabetes is associated with a more severe disease progression and higher mortality risk [45,46,47,48,49,50]. The mechanisms linking diabetes to SARS-CoV-2 are various. The immunosuppressive state associated with diabetes could make diabetic patients more susceptible to 2019-nCoV infection [51,52]. In fact, diabetes induces alterations in innate immunity, impairing phagocytosis, neutrophil chemotaxis and innate cell-mediated immunity [52,53]. Diabetes is even associated with a pro-thrombotic state, which predisposes to thrombotic complications [54]. Moreover, diabetic patients have lower levels of ACE 2. As previously mentioned, lower expression of ACE 2 correlates with reduced levels of Ang 1-7 and increased levels of Ang II, leading to a more severe illness [55]. Interestingly, a study by Yan et al. showed that 24.9% of 193 patients with severe SARS-CoV-2 had diabetes. Diabetic patients had higher levels of leukocytes, neutrophils, c-reactive protein, procalcitonin, ferritin, IL-2 receptor, IL-6, IL-8, TNF-α, D-dimer, fibrinogen, lactic dehydrogenase, troponin I and NT-proBNP, reflecting a more severe inflammatory response and a higher risk for severe SARS-CoV-2 compared with patients without diabetes [46].

In addition, ACE 2 in islet pancreatic cells could determine transient insulin dependent hyperglycemia linked to a direct viral injury in the pancreas [45,53].

Because cardiovascular comorbidities are significant predictors of severe disease and mortality, an early identification of these conditions is essential to ameliorate management and to improve SARS-CoV-2 prognosis.

### 4.2. SARS-CoV-2, Cardiovascular Manifestations and Mechanisms of Injury

Different studies showed that cardiovascular injury is associated with 2019-nCoV infection [40,56] and its outcomes [39,56,57,58], with arrhythmias, cardiomyopathy and cardiac arrest as possible terminal events of the disease [24].

In particular, Shi et al. showed, in a retrospective cohort study, that 19.7% out of 416 SARS-CoV-2 patients had cardiac injury [39]. Among them, 14 patients showed, at the electrocardiogram after admission, abnormalities―in particular T-wave depression or inversion, ST- segment depression and Q waves―compatible with myocardial ischemia. They required even more often non-invasive ventilation and invasive mechanical ventilation and had a higher mortality rate than the control group. Guo et al. showed that higher levels of troponin I and cardiovascular comorbidities correlated with a higher mortality [58]. Moreover, Lippi et al. confirmed in a meta-analysis of four studies that elevated values of troponin I correlated with severe disease [57]. Additionally Zhou et al. showed that heart failure [23%] and acute cardiac injury [17%] were common complications of SARS-CoV-2 [40]. A study by Gao et al. demonstrated that patients with higher NT-proBNP levels have increased systemic inflammation and cardiac injury markers; therefore, higher NT-proBNP levels can predict in-hospital death [59]. Taken together, the evidences discussed above suggest that screening for myocardial injury with an electrocardiogram at time of hospital admission and determination of myocardial markers to identify patients at risk for unfavorable disease evolution, hemodynamic instability and shock, can be useful for risk stratification and prevention of complications [58].

Cardiovascular manifestation linked to SARS-CoV-2 are various. The disease could present with myocardial infarction. In particular, a retrospective study by Stefanini et al., on 28 patients with SARS-CoV-2, demonstrated that ST-segment elevation myocardial infarction [STEMI] was the first sign of infection in 85.7% of cases. Among them, 60.7% of patients demonstrated culprit lesions at the coronary angiography, while 39.3% of patients did not present a coronary obstruction [60], suggesting a type 2 myocardial infarction. In a case report by Ueki et al., a patient with SARS-CoV-2, fever and mild dyspnoea, showed acute right-sided pulmonary embolism at CT-scan and signs of acute infero-posterior STEMI at the electrocardiogram, in absence of chest pain. Coronary angiography demonstrated a partial stenosis of the circumflex artery with a thrombus on a lipid-rich plaque, in absence of plaque rupture [61]. Even Bangalore et al. described a case series of 18 patients with 2019-nCoV infection and ST-elevation on electrocardiography [62]. Among these patients, 9 underwent coronary angiography of which 67% had a coronary artery stenosis [62].

Different mechanisms are implicated in the pathogenesis of SARS-CoV-2 and cardiac ischemic injury. An acute inflammatory response could promote endothelial dysfunction with an increased risk of pre-existing coronary atherosclerotic plaque rupture [39]. Additionally, inflammation is associated with hypercoagulability, which promotes occlusive thrombi formation [39]. Patients with severe SARS-CoV-2 show high levels of IL-2, IL-7, IL-10, granulocyte-colony stimulating factor [G-CSF], CXCL10, MCP-1, macrophage inflammatory protein 1-α, and TNF-α [6,63], which could enhance endothelial dysfunction and acute thrombosis, as shown in a case series of patients with severe SARS-CoV-2 and venous thrombosis or arteriosclerosis obliterans of lower extremities [64]. SARS-CoV-2 is even associated with other hemostasis disturbances, particularly with mild thrombocytopenia and increased D-dimer levels, which correlate with a severe illness; in addition, a correlation with prolonged prothrombin, thrombin and activated partial thromboplastin time was demonstrated [65,66]. It remains unclear if these abnormalities are due to a direct virus effect or an indirect cytokine storm effect caused by the virus. Moreover, hemostasis abnormalities noted in SARS-CoV-2 could be a result of liver dysfunction related to the infection [65]. Interestingly, a case series by Varga et al. including SARS-CoV-2 patients who died, demonstrated that 2019-nCoV directly injures endothelial cells, leading to a microvascular damage sustained by a diffuse endothelial inflammation [67]. Another mechanism, involved in cardiac ischemic damage correlated with SARS-CoV-2, is related to hypoxia due to lung injury. This may impair oxygen demand leading to myocardial ischemia, resulting in a type 2 myocardial infarction [24,68].

Cardiovascular injury may manifest even with rhythms disturbances. Arrhythmias of unknown origin were demonstrated in 16.7% of 138 SARS-CoV-2 patients hospitalized at the general ward and 44% of 138 patients admitted to the intensive care unit [56]. Disturbance in cardiac rhythm may be due to hypoxia and inflammation, which are associated to SARS-CoV-2 pneumonia [69]. In addition, medical therapy like chloroquine, hydroxychloroquine, lopinavir/ritonavir and azithromycin, used in the setting of SARS-CoV-2, could exacerbate arrhythmias, leading to corrected QT prolongation [69,70]. Furthermore, chloroquine and hydroxychloroquine, acting on intracellular pH, may promote atrioventricular block and torsade de pointe, and may affect electrolyte balance. In addition, the two drugs could increase risk of bradycardia, PR prolongation and atrioventricular block inhibiting CYP2D6, which increases exposure to beta blockers [70].

Cardiomyopathy and myocarditis are other manifestation of cardiovascular involvement; Hendren et al. recognized a cardiovascular syndrome characterized by acute myocardial injury, cardiomyopathy and arrhythmias, in absence of coronary artery disease [71]. In a case series by Arentz et al., 33% of 21 patients with SARS-CoV-2 developed cardiomyopathy, even if it is unclear if myocardial injury was a direct effect of the virus or a complication of the infection [72]. In particular, two different mechanisms have been hypothesized in the pathogenesis of myocardial injury linked to SARS-CoV-2. The first is a direct effect of the virus on cardiomyocytes with the development of a myocarditis or a stress cardiomyopathy [73]. The second is an indirect effect of imbalanced T helper 1 and T helper 2 responses determining an intense cytokine release; this could cause or contribute to cardiac dysfunction, leading to apoptosis and necrosis of cardiomyocytes, increased vascular wall permeability and edema [19,39,71]. In fact, patients with SARS-CoV-2 admitted to the intensive care unit have higher serum cytokine and chemokine levels [6,63]. Cardiac damage, due to systemic cytokines release, is common even in other conditions. In particular, chimeric antigen receptor [CAR]-T cell therapy could trigger a cytokines release syndrome characterized by acute myocardial injury. In this setting, administration of tocilizumab, an IL-6 inhibitor, post-CAR-T is associated with a lower rate of cardiovascular events [74].

During the management of SARS-CoV-2, even drug-drug interactions should be considered as precipitating factors of cardiovascular damage. In fact, lopinavir/ritonavir, two combined protease inhibitors used to treat HIV and nowadays SARS-CoV-2, inhibit bioactivation of prasugrel and clopidogrel, reducing antiplatelet activity [65,70].

Interestingly, myocarditis associated with 2019-nCoV infection in patients without history of cardiovascular disease was reported in literature [75,76,77,78]. Myocarditis develops around two weeks after the onset of 2019-nCoV infection and is clinically characterized by mild chest pain or discomfort and palpitations [9], with transient abnormalities on the electrocardiogram, such as atrioventricular block and tachyarrhythmias and left ventricular function abnormalities [9]. Magnetic resonance can give hints on myocardial injury and endomyocardial biopsy is used to confirm the diagnosis. Until now, a clear cardiac tropism of 2019-nCoV is not demonstrated. In fact, Tavazzi et al. described a case of acute myocarditis with finding of viral particles only in interstitial cytopathic macrophages [78]. Moreover, in a study conducted on 12 consecutive autopsies of SARS-CoV-2 patients, viral RNA was found in the hearts of five patients, even though the exact localization was not specified [79]. An interesting case report, by Sala et al., described a case of myocardial dysfunction with reverse Tako-Tsubo syndrome detected at coronary CT angiography and cardiac magnetic resonance, with a successive recovery of systolic function [80]. Moreover, authors described T-lymphocytic inflammatory infiltrates with interstitial edema and foci of necrosis present at endomyocardial biopsy; the SARS-CoV-2 genome was not found in the examined tissue [80]. Interestingly, other cases of the Tako-Tsubo syndrome are reported in SARS-CoV-2 patients [81,82,83,84]. The Tako-Tsubo syndrome is a stress-related cardiomyopathy that can be triggered by emotional or physical stress. It is characterized by transient left ventricular apical wall motion abnormalities due to coronary artery vasospasm, microvascular dysfunction and catecholamine toxicity [85]. Stress-induced adrenergic response caused by fever, inflammation and microvascular viral injury could play a role in the pathogenesis of SARS-CoV-2 stress cardiomyopathy; however, additional researches are needed to elucidate the association between 2019-nCoV infection and Tako-Tsubo syndrome.

## 5. Treatment

A specific treatment for SARS-CoV-2 has not been identified yet. Chloroquine and hydroxychloroquine―immunomodulant drugs used in the setting of malaria and autoimmune diseases―reduce viral entry into cells by increasing endosomial pH and interference with glycosylation of ACE 2 [86]; in SARS-CoV-2 patients, they seem to reduce disease severity and hospitalization time [87,88]. The interaction between 2019-nCoV and ACE 2 proposes that ARBs could reduce risks and SARS-CoV-2 severity. Particularly, losartan is a promising drug because of its capacity to upregulate ACE 2, which prevents Ang II production implicated in lung injury [89,90]. However, the effect of RAS blockade in SARS-CoV-2 patients remains unknown [91]. Recombinant human ACE 2 is assessed for its potential ability to neutralize viral particles and to reduce levels of Ang II and IL-6, and Camostat mesylate, a serine protease inhibitor that stops TMPRSS2 activity, could block SARS-CoV-2 entry into cells [73].

The use of corticosteroids in the setting of SARS-CoV-2 is still matter of debate. Until now, the World Health Organization consensus document on the management of SARS-CoV-2 does not encourage corticosteroids use [92]. Some authors suggest steroids only in the late-stage of ARDS, because of delayed virus clearance risk in the early phase of infection [93]. A study by Wang et al. evaluated the effect of methylprednisolone on 46 severe SARS-CoV-2 patients showing that the use of the corticosteroid reduced mechanical ventilation risk, supplemental oxygen therapy intervals and hospitalization length in intensive care units [94]. Moreover, a preliminary study by Horby et al. described the effect of dexamethasone compared with standard care, showing that the treatment with dexamethasone reduced the incidence of death in patients receiving invasive mechanical ventilation and supplemental oxygen without invasive mechanical ventilation but not in those not receiving respiratory support [95].

Among antiviral therapies, lopinavir/ritonavir have been evaluated to treat SARS-CoV-2. Lopinavir is an aspartate protease inhibitor used to treat HIV, and was identified as an available treatment against SARS-CoV and MERS-CoV, reducing adverse clinical outcomes. Ritonavir combined with lopinavir increases its plasma half-life [96]. However, a recent randomized, controlled, open-label trial by Cao et al. showed no treatment benefit with lopinavir/ritonavir respect the standard care [96].

Remdesivir is an adenosine analogue, which acts against RNA viruses and in vitro it seems effective controlling 2019-nCoV infection [97,98]. Tocilizumab is a monoclonal antibody used in the setting of different autoimmune diseases directed against IL-6 receptors, which downregulates the excess inflammatory response. As mentioned, severe SARS-CoV-2 is characterized by a cytokines storm and tocilizumab seems to reduce clinical severity of the infection [96,99,100]. However, several studies assess treatment effectiveness in SARS-CoV-2 patients [92]. Cell therapy, to treat SARS-CoV-2, is currently as well assessed. In particular, the role of mesenchymal stem cells [MSCs], used in immune-mediated inflammatory diseases, able to reduce over activation of the immune system, has been evaluated in a work by Leng et al. on 7 SARS-CoV-2 patients, which showed improved lung function two days after transplantation [101].

Furthermore, Amdekar et al. studied the effectiveness of cardiosphere-derived cells [CDCs] [102]. CDCs are stromal progenitor cells, isolated from human heart tissue and have anti-inflammatory properties, targeting proinflammatory cytokines like TNF-α, IFN-γ, IL-1β, and IL-6, and anti-inflammatory pathways [30]. Amdekar et al. showed, in a case series of 7 critical ill patients, that intravenous infusion of CDCs was well tolerated and associated with the recovery of four patients [102]. Given the interesting results of these studies, additional works are needed to assess the efficacy of cell therapy on SARS-CoV-2.

Finally, treatment with coalescent plasma is being evaluated. In fact, immunoglobulins of SARS-CoV-2 from recovered patients could improve the disease course through viral neutralization.

At this time, a definitive treatment for SARS-CoV-2 infection is not available; therefore, preventive measures are of utmost importance.

In Table 1 are reported the most relevant studies regarding the relationship between SARS-CoV-2 infection and cardiovascular diseases.

## 6. Conclusions

SARS-CoV-2 is a global pandemic and host immune response plays a pivotal a role in determining the severity of illness. Patients with cardiovascular disease are at high risk to develop a more severe illness and cardiovascular risk assessment is mandatory in all infected patient, because of disease prognosis. Regular clinical examination, electrocardiograms and laboratory exams are needed to early detect cardiovascular complications, such as myocardial ischemia, heart failure and arrhythmias. Given currently available evidences, continuation of RAS blockers is recommended and drug-drug interactions must be carefully monitored. However, further research is needed to clarify the role of 2019-nCoV in myocardial injury and to develop an effective treatment strategy. Moreover additional studies will be useful to elucidate the role of immune response and clinical manifestations of 2019-nCoV infection, broadening the spectrum of therapeutic options.

## Figures and Tables

**Table 1:** Studies showing a relationship between SARS-CoV-2 infection and cardiovascular disease.

Authors	Study Design	No. of Patients	Cardiovascular Involvement
[1]	Retrospective, multicentre study	1099	Comorbidity [2.5% of patients with CAD]
[6]	Retrospective	41	Comorbidity [15% of patients with CVD] and complication [12% of patients with acute cardiac injury; 31% admitted to ICU, 4% non ICU]
[24]	Retrospective, multicentre study	8910	Comorbidity associated with mortality [20% of patients non survivors with CAD, 5.6% with HF, 6.8% with arrhythmia]
[39]	Retrospective study	416	Complication associated with mortality [19.7% of patients with cardiac injury, mortality in patients with cardiac injury 51.2%]
[40]	Retrospective, multicentre study	191	Comorbidity [8% of patients with CAD] and mortality [24% of patient with CAD]
[41]	Retrospective, multicentre study	355	Comorbidity [30% of patients with ischemic heart disease, 24.5% of patients with atrial fibrillation]
[42]	Retrospective study	99	Comorbidity [40% of patients with CV and CBV diseases]
[56]	Retrospective case series	138	Comorbidity [14.5% of patients with CVD]. Complication [44.4% of patients admitted to ICU
[46]	Retrospective study	193	Comorbidity [16.1% of patients with CVD, 27.1% of diabetic patients with CVD] and mortality [25% of patients non survivors with CVD].Diabetes
[47]	Retrospective study	174	Comorbidity [32.4% of diabetic patients with CVD].
[49]	Retrospective study	28	Comorbidity and outcome [ICU patients have higher levels of Troponin I]
[58]	Retrospective case series	187	Comorbidity [35.3% of patients with CVD, 27.8% of patients with myocardial injury] and mortality [13.33% of patients with CVD, 37.5% of patients with elevated levels of Troponin I, 69.44% of patients with CVD and elevated levels of Troponin I]
[60]	Retrospective study	28	Complication [STEMI as first presentation in 85.7% of cases]
[63]	Retrospective study	21	Complication [9.1% of patients with acute cardiac injury]
[59]	Case series	54	NT-proBNP associated with higher mortality
[72]	Case series	21	Comorbidity [42.9% of patients with congestive heart failure]. Complication [33.3% of patients develop cardiomyopathy]
[62]	Case series	18	Complication [STEMI]
[61]	Case report	1	Complication [STEMI]
[75]	Case report	1	Complication [myocarditis]
[76]	Case report	1	Complication [myocarditis]
[77]	Case report	1	Complication [myocarditis]
[80]	Case report	1	Complication [myocarditis]
[78]	Case report	1	Complication [myocarditis]
[81]	Case report	1	Complication [Tako-Tsubo syndrome]
[82]	Case report	1	Complication [Tako-Tsubo syndrome]
[83]	Case report	1	Complication [Tako-Tsubo syndrome]
[84]	Case report	1	Complication [Tako-Tsubo syndrome, atrial fibrillation]

## Abbreviations

CAD	coronary artery disease
CVD	cardiovascular disease
ICU	intensive care unit
HF	heart failure
CBV	cerebrovascular
